# Ethical and policy issues raised by uterus transplants

**DOI:** 10.1093/bmb/ldz022

**Published:** 2019-09-02

**Authors:** Laura O’Donovan, Nicola Jane Williams, Stephen Wilkinson

**Affiliations:** Department of Politics, Philosophy, & Religion, County South, Lancaster University, Lancaster, LA1 4YQ, United Kingdom

**Keywords:** access to treatment, AUFI, consent, donors, ethics, funding, infertility, reproduction, transplantation, uterus

## Abstract

**Introduction:**

In 2014, Brännström and colleagues reported the first human live birth following uterine transplantation (UTx). Research into this treatment for absolute uterine factor infertility has since grown with clinical trials currently taking place across centers in at least thirteen countries worldwide.

**Sources of data:**

This review summarizes and critiques the academic literature on ethical and policy issues raised by UTx.

**Areas of agreement:**

There is general agreement on the importance of risk reduction and, in principle, to the sharing and maintenance of patient data on an international registry.

**Areas of controversy:**

There are numerous areas of controversy ranging from whether it is ethically justified to carry out uterus transplants at all (considering the associated health risks) to how deceased donor organs for transplant should be allocated. This review focuses on three key issues: the choice between deceased and living donors, ensuring valid consent to the procedure and access to treatment.

**Growing points:**

UTx is presently a novel and rare procedure but is likely to become more commonplace in the foreseeable future, given the large number of surgical teams working on it worldwide.

**Areas timely for developing research:**

Uterus transplantation requires us to re-examine fundamental questions about the ethical and social value of gestation. If eventually extended to transgender women or even to men, it may also require us to reconceptualize what it is to be a ‘father’ or to be a ‘mother’, and the definition of these terms in law.

## Introduction

Research into uterine transplantation (UTx) dates back to 1960. However, it was not until 2000 that the first modern attempt at human UTx took place, in Saudi Arabia. Though unsuccessful, clinical research by various groups gathered pace and, in 2014, Brännström and colleagues in Sweden reported the first live birth following living-donor UTx.^1^ To date, there have been 11 reported live births following living-donor transplant and 2 following deceased-donor transplant.^2^ In 2015, the National Health Service (NHS) Health Research Authority granted approval for a UK trial programme at Hammersmith Hospital involving 10 patients and brain-stem dead, heart-beating donors.^3^ More recently, in 2018, it was announced that this study would also include five transplants from living donors.^4^

Absolute uterine factor infertility (AUFI) is the absence of a functional uterus^5^ due to congenital Müllerian malformations or acquired causes and is described by Brännström as ‘the only major type of female infertility still viewed as untreatable’.^1^ Approximately 1 in 500 women worldwide are estimated to have uterine factor infertility,^6^ with around 15 000 women of childbearing age in the UK having no womb.^7^ The current options for women with AUFI who wish to have children are adoption or surrogacy. However, both can be lengthy, bureaucratic and expensive processes and, while altruistic surrogacy is legally permitted in the UK, not everyone considers it an acceptable option (for cultural, moral or practical reasons). UTx, on the other hand, can provide women with the genetic, gestational, legal and social components of motherhood without the need for reliance on a surrogate, and also offers the experience of having a child as a result of one’s own pregnancy.

UTx is the world’s first ‘ephemeral’ transplant with most study models recommending hysterectomy after a certain period. The treatment finds itself at the cutting edge of science, occupying the middle ground between innovative transplantation and developments in assisted reproductive technology (ART). Because of this, a wide-ranging ethical and legal literature has arisen in a short space of time. The issues it addresses may usefully be categorized into the broad themes of: transplantation ethics; donation ethics; questions of access; child welfare; and ethical research design and practice. This review explores three particular concerns that have attracted most interest: the value of gestation, the choice between deceased or living donors and access to treatment.

### Areas of agreement in principle

The risk of physical and psychological complications to recipient, live donor and resulting child have occupied a large portion of the literature to date. The first modern attempt at human UTx unfortunately resulted in acute vascular thrombosis requiring removal 99 days after transplantation.^8^ Duration of surgery has also been a cause for concern with research ongoing into the use of robotic-assisted surgery with the aim of reducing the operative time for donors and recipients.^9^^,^^10^ Further physical health risks include post-operative complications such as infection, thrombosis, fistula and uretic injury^11^ and psychological risks include issues relating to gender identity and sexual dysfunction. There are also more general risks such as complications arising from immunosuppressive therapy and psychological problems resulting from transplant surgery. Acknowledging this, there is a consensus that uterus transplants should not be offered as part of routine clinical practice until safety and efficacy are proven.^12^ More data are required in order to fully understand the risks treatment poses and, at this stage, limiting UTx is preferable while ongoing trial outcomes are explored.

The second area of agreement in principle concerns the importance of recording and maintaining data accumulated as part of registered trials worldwide ‘to further optimize the procedure concerning efficiency and safety’.^13^ The operation of such a registry not only permits data sharing on outcomes enabling safety monitoring, but also provides a mechanism through which the practice of UTx can be regulated.^14^ Although there may be broad consensus that data should be shared, the extent of the information included and the principles governing data use (EU countries are now subject to General Data Protection Regulations) pose further questions for debate moving forward.

### The value of gestation

The value of gestation is a major theme in the ethics literature on UTx. Two main concerns emerge in the various discussions: the extent to which UTx serves to reinforce social biases regarding reproduction, exacerbating the harm caused by infertility, and whether providing UTx causes alternative options to be less acceptable or desirable. Much criticism of ART relates to concerns about the ‘motherhood mandate’, an ideology according to which motherhood is central to female identity and ‘having at least two children and raising them well’ is a norm or requirement for adult women.^15^ While advances in medicine provide women with more reproductive choice, commentators have expressed concerns that increasing options, reflective of prevailing social and cultural norms, may intensify both the strength of the desire to procreate using ARTs and the harm suffered by individuals who cannot or choose not to do so.^16^ ARTs (perhaps especially UTx) arguably promote a particular kind of family, the biological nuclear family, in which the recipient will be both the genetic and gestational mother of any child born. However, a society in which biological ties are less valorized may be beneficial and ameliorate some of the harms caused by infertility. A related concern is that ARTs, such as UTx, promote and perpetuate the dominance of the traditional and/or genetic family described above; as this is not an option for some women, their distress will be worsened if they are made to feel that surrogacy, adoption or voluntary childlessness are inferior alternatives.^16^^,^^17^

### Living versus deceased donation

Around 75% of UTx procedures (34/45 reported cases) have utilized living donors, the majority of which (24/34) have been close relatives of the intended recipients (mothers, aunts and sisters) with only 25% (11 reported cases) using uteri from deceased brain-dead donors. As with other transplants where both living and deceased donor organs are available, each model comes with distinct benefits and challenges. Significant debate has thus arisen about the weight assigned to each and thus to the question of which model should be preferred assuming that both are eventually proven sufficiently safe and effective.

#### Clinical and practical issues

One key factor influencing the choice of donor model is the clinical and practical benefits and challenges associated with each. Given the relatively small quantity of transplants performed so far, and the number of variables influencing success rates, it is difficult to be certain at present about the relative merits of each. The deceased donor model for UTx has been associated with several possible benefits. These include the ability to retrieve longer lengths of vasculature (which may reduce the risk of serious complications for recipients seen in the living donor model such as thrombosis) and a simplified transplantation procedure that reduces surgical time and risks of anaesthesia in recipient surgeries.^18^ However, despite such benefits, the majority of physicians trialling UTx hold that—as in other living donation contexts, such as kidney donation and liver lobe donation—the living donor model is liable to provide greater benefits. Whether this is the case will become clearer as trials progress, but benefits include: closer tissue matching where relatives are used; higher organ quality due to significantly lowered warm and cold ischemia times; reduced likelihood of transplanting a uterus that is unsuitable for gestation and/or diseased due to the ability to conduct thorough testing prior to retrieval absent significant time pressures; the ability more easily to schedule complex surgeries including a large team of physicians from different specialties; and reduced waiting times resulting from deceased donor organ scarcity.^19^^,^^20^

#### Ethical considerations

While a concern to maximize success rates and provide practical benefits leads to a preference for living donors, a concern for the welfare of and to respect the autonomy of donors tends to pull in the opposite direction. In terms of welfare, for example, while the deceased model poses no risks to donors, living donation both necessitates and risks serious harms,^21^^,^^22^ thought to be similar to, or slightly greater than, those associated with a total abdominal hysterectomy.^23^ These are likely to diminish over time as surgical techniques and post-operative care are finessed. However, of the cases reported so far, four living donors have experienced significant complications requiring surgical, endoscopic or radiological intervention under anaesthetic^24^^,^^25^^,^^26^^,^^27^ and other donors have experienced infections, urinary hypotonia, leg and buttock pain, and depression.^28^ Similarly, concerns have also been raised about the welfare of living donors who may end up regretting their choice to donate—should, for example, the retrieval/transplant go awry, their relationship with the recipient sour, or they find themselves wanting pregnancy later in life—which, again, is avoided in cases where deceased donors are used.^29^ Some commentators, however, have noted that, while deceased donors in UTx are not at risk of harm, their use does pose risks of harm to third parties in certain contexts. Most notable of these is the risk to vital organs (ones which would otherwise be available for transplantation) that could occur if uteri were retrieved prior to life-saving organs.^30^

In terms of respect for autonomy, it could simply be argued that permitting living donation respects the autonomy of those who wish to donate by allowing them to do so. However, the position is actually more complex. For both living and deceased donation, there is a risk of uteri being obtained without *sufficiently high-quality consent*. For living donation, concerns center around the possibility of living related donors experiencing external pressures such as coercion or manipulation to donate from the recipient or other family members,^8^ and that living unrelated donors may be offered incentives (financial or otherwise) that could constitute an autonomy-undermining inducement.^31^ The design and implementation of robust consent procedures can reduce such risks, as in other donation contexts. However, some risks will always remain and will be higher in lower income and more pro-natalist societies.^32^ In the context of deceased donation, the unfamiliarity of UTx combined with its quality of life-enhancing purpose, also poses challenges for obtaining appropriate consent from donors and/or their families post-mortem. Given these considerations, consent to donate a uterus cannot necessarily be inferred from an individual’s possession of a donor card^33^ and family members may find it difficult to reach an informed decision about the deceased person’s preferences.^34^^,^^35^ However, unlike in the context of living donation, these concerns both pose no threat to the psychological welfare of donors and are likely significantly to decrease over time if uterus transplantation reaches public consciousness and becomes included in donor registration lists.

#### A balancing act

Given the risks and harms associated with living donation, a small minority of scholars suggest that—as a result of the physician’s duty of non-maleficence and UTx’s status as a quality-of-life, as opposed to life-saving, transplant—living donation is inappropriate. The majority, however, take a more moderate stance suggesting instead that, while harm and risk to donors offer *some* reason to prefer uteri from deceased donors, this must be balanced against the benefits it offers.^36^ Thus, as in other donation contexts, living donation can be justified provided that

- valid and informed consent is given by the donor after mandatory and in-depth counseling from donor physicians and psychologists;
- levels of harm suffered by donors are both proportionate to the benefits produced and fall below some accepted threshold; and,
- attempts are made to minimize the use of living donors and any harms inflicted to them.^34^

Harm minimization may be achieved through, for example:

- the promotion of alternatives to transplant such as surrogacy and adoption (where permitted)^37^;
- the use of living donors who have already completed their families/are undergoing removal of healthy uteri as part of a wider gynecological procedure or gender affirmation surgery^38^;
- expansion of the deceased donor pool to include increased/non-standard risk donors^39^; and/or,
- supporting research into future advances in ART which may render the use of living donors obsolete such as the bioengineered uterus.^34^

### Access to UTx

Three main issues arise when considering access to UTx programmes:

(1) Should treatment be publicly funded?
(2) What selection criteria should be adopted to define the eligible patient base?
(3) What factors should be incorporated into the allocation ranking system to ensure the equitable distribution of non-directed donor uteri?

#### Should treatment be publicly funded?

In the UK, the question of funding this procedure UTx will fall to NHS commissioners once a sufficient evidence base has been established. In April 2017, UTx was added to the list of prescribed specialized services for which NHS England is the responsible commissioner.^40^ This avoids clinical commissioning groups having to make difficult decisions in respect of local budgets where significant differences in the provision of standard treatment for infertility (including in vitro fertilization (IVF) and intracytoplasmic sperm injection (ICSI) exist through out UK.^41^

Ethical opinion on the public funding of UTx is divided. Some critics oppose it altogether citing arguments^42^ relating to the existence and promotion of alternative options (such as surrogacy or adoption)^16^ and questioning the extent to which UTx both responds to, and reinforces, a socially-conditioned desire to reproduce in a particular way.^17^^,^^43^ To this end, commentators have argued that ‘the rhetoric surrounding uterine transplantation points to connections between the ability to experience gestation and womanhood or femininity’.^17^ This may result in devaluing other modes of family formation in light of prevailing social biases: pronatalism and geneticism.^16^^,^^17^ However, these arguments also apply to assisted reproduction more generally. For example, the desire to undergo IVF in order to have a genetically related child potentially raises the same concerns about pronatalism and geneticism and yet publicly funded IVF has not thus far been refused on these grounds. A stronger argument against funding can be found in discussions of the risks of treatment to recipient, donor and prospective child.^44^ In this respect, UTx is presently in a weaker position than other ARTs, because it has a less well-established track record of safety and efficacy.^45^

Objections to funding UTx have been challenged by various commentators who emphasize the disease status of infertility (notwithstanding the possibility that this may be exacerbated by people’s socially conditioned desires for particular family forms^37^^,^^46^) and the difficulty of evaluating the ‘sufficiently good alternatives’ to UTx.^37^ Whether surrogacy or adoption are more or less ‘valuable’ than UTx depends in part on the personal preferences of prospective patients.^37^ Where experiencing gestation is significant for the individual concerned and both safety and efficacy of UTx practice are proven, it may be the case that public funding is justified in the interests of patient autonomy and wellbeing, if the wider social and psychological context is taken into account.^47^

#### Selection and allocation criteria

UTx generates particular issues regarding the selection criteria applicable to prospective patients and the allocation criteria employed in order to establish a fair system of organ distribution. As with other ART services, it seems sensible to employ patient selection criteria in order to ensure that only those patients with realistic prospects of success enter treatment. In the context of UTx these have included requirements such as

- being genetically female;
- being able to provide one’s own oocytes and/or embryos.
- being able to demonstrate child-rearing capacity; and
- having appropriate reasons for seeking treatment.

All clinical trials to date have insisted that the recipient should be a genetic female with no medical contraindications to transplantation.^48^ For the UK clinical trial, this is expressed as a requirement that the recipient has normal ovarian reserve and function.^14^ It has been suggested that there may be a case for eventually providing UTx to transgender women, enabling the alignment of reproductive capabilities with acquired gender identity.^49^ However, due to anatomical and physiological differences between chromosomally male and female bodies, further scientific research is required in order to demonstrate feasibility^50^ and, as such, the exclusion of chromosomally male recipients from UTx trials seems justified for the time being.

Criteria relating to child-rearing capacity are also contentious. Commentators have argued that, given that the purpose of UTx is childbirth, recipients should be required to meet certain quality thresholds for child-rearing.^34^^,^^51^ Criminal background checks, as well as financial and socio-psychological evaluations have been proposed to assess comprehensively parenting ability.^34^ In the UK, this is encapsulated to a less invasive extent in the ‘welfare of the child’ assessment required by the Human Fertilisation and Embryology Act 1990 (as amended) *prior* to the provision of any treatment services. This applies to all fertility treatment regulated by the Human Fertilisation and Embryology Authority and as such is better understood as a threshold selection requirement. Clinicians must take account of the welfare of any child who may be born as a result of the treatment (including the need of that child for supportive parenting) and of any other child who may be affected by the birth.^52^ Common considerations include ‘any aspects of the patient’s (or their partner’s) (a) past or current circumstances that may lead to any child experiencing serious physical or psychological harm, or neglect, or, (b) circumstances that are likely to lead to an inability to care throughout childhood for any child born as a result of treatment, or that are already seriously impairing the care of an existing child of the family’.^53^ While consideration of the welfare of the future child in ART treatment is accepted (though not without controversy^54^), commentators have been careful to warn against biased social value judgments, particularly regarding questions as to what might constitute a ‘good mother’,^34^ in order to avoid unfair discrimination between prospective patients.

Consideration of patients’ reasons for seeking treatment is relatively uncontroversial; it is generally agreed that treatment should only be provided to those seeking UTx in order to reproduce and become mothers and not merely to restore or acquire bodily function.^34^

Similar discussions have arisen regarding equitable organ allocation policy. Not only is UTx a quality of life enhancing as opposed to life-saving transplant, it also presents distinct and complex allocation challenges. In part, this is because AUFI in particular does not come in degrees (because all those with it have an equal chance, i.e. no chance, of reproducing ‘naturally’). Therefore, factors additional to ones traditionally applied in the context of other composite tissue allografts (assessing clinical health status) are required to ensure the fair allocation of non-directed donor uteri. Due to the stated goal and purpose of UTx, social factors will be important in any proposed patient ranking system. To this end, commentators^34^^,^^51^ have suggested criteria based on a variety of psychosocial and medical factors, such as

- presence of existing children, especially where these are biologically related to the prospective recipient;
- amount (and cost) of infertility treatment required;
- priority for those for whom it is hard to find a suitable donor (e.g. highly sensitized groups, those with high antibody levels, members of some ethnic minority groups); and,
- age of the recipient.

Regarding the presence of existing children, it has been suggested that women who have already experienced gestation and birth or, more strongly, are already genetic and/or social parents should receive lower priority.^34^^,^^51^ In publicly funded healthcare systems, the presence of an existing child of the patient or of the family is sometimes utilized (for example, by Clinical Commissioning Groups in England) as a threshold criterion to limit access to available services.^41^ Given finite resources, this kind of ranking may be justified in order to ensure fair treatment distribution.^34^ However, any proposal to limit access to those who have not experienced gestation on grounds of current parenthood is liable to prove controversial.

As a criterion to assist with patient ranking, the amount of infertility treatment required is currently of limited assistance. All patients will require IVF given that the uterine graft is not connected to the patient’s fallopian tubes, and that therefore natural pregnancy is not possible. However, if further clinical research enables natural pregnancy following UTx in future, prioritizing women requiring transplant only could potentially be justified on cost-effectiveness grounds.^51^ Further, prioritizing those who are members of highly sensitized groups due to difficulties associated with finding suitable tissue matches is uncontroversial. This is routinely considered in the allocation of solid-organ transplants where it does not prejudice severely ill patients, and should similarly apply to UTx.

Ranking criteria based on the age of the recipient also provide an interesting area for discussion. The purpose of UTx is to provide or restore reproductive capacity to women with AUFI. Reproductive function naturally declines with age and so it is argued that ranking policies ought to mimic the natural reproductive lifecycle and seek to admit only those women falling within an ‘age-relative opportunity range’.^51^ Views on what constitutes normal childbearing age will vary between countries and, due to differing social norms and practices, this may be an area where it is difficult to reach international agreement. In the US literature, account has been taken of definitions advanced by the World Health Organisation, assessment of the medical literature on advanced maternal pregnancy and egg donation, and consideration of the hardships of teen pregnancy, resulting in a proposed range of between 20 and 45 years. In publicly-funded systems like the NHS, it is important to ensure equality between the different assisted conception treatments, particularly given that UTx patients also require standard IVF. Guidelines from the National Institute of Health and Care Excellence state that women up to age 40 should be offered three full cycles of IVF, while women aged 40–42 years should be offered one. This suggests an upper age limit of 42 for UTx in the UK, though current recipient inclusion criteria in the UK clinical trial mandates an age range of 24–38 (40 if embryos frozen <38 years).^14^ Further account then ought to be taken of where women fall within this established age range. It has been suggested that those nearing the upper limit ought to be afforded additional priority on the basis of their retreating opportunity to have children.^34^^,^^51^ It has also been suggested that time on the waiting list should be considered.^34^^,^^51^ How much weight this should be accorded in the ranking process is a question that requires further work, especially considering the fact that women with AUFI due to congenital abnormality will be able to seek to join the waiting list from the lower age limit.

### The way forward

Uterus transplantation raises important ethical, social, and regulatory questions. Some of these result from, or at least are exacerbated by, the experimental status of UTx. For these, answers or solutions are likely to emerge over the short- to medium-term as better data about the benefits and risks of UTx emerge, and policy is amended in the light of these. This applies in particular to issues such as whether to use living or deceased donors; the welfare of children born through UTx; whether UTx meets funding thresholds; UTx in transgender populations; and allocation criteria for recipients. However, uterus transplantation also forces re-examination of more foundational questions, ones that cannot be fully answered by trial data. These are ultimately questions of axiology, of what we do and should value, and how we should respond to that value individually and as a society. Such questions include but are not limited to:

- What value should we ascribe to gestation and to enabling people to carry their own future children within the womb?
- What responsibilities as a society do we have to alleviate the social and psychological harms caused by infertility?
- What are the proper limits of medicine?
- What levels of risk to donors and recipients are acceptable for quality of life enhancing (as opposed to life-prolonging) transplantations?
- What resource priority should infertility treatment services be assigned compared to other health-related interventions?

## Funding

This work was supported by the Wellcome Trust grant number 097897/Z/11/Z and The Leverhulme Trust grant number ECF-2018-113.

## Conflict of interest statement

The authors have no potential conflicts of interest.

## References

[1] Brännström M, Johannesson L, Bokström H, et al. Livebirth after uterus transplantation. The Lancet 2015;385:607–16. 10.1016/S0140-6736(14)61728-1 25301505

[2] Ejzenberg D, Andraus W, Baratelli Carelli Mendes LR, et al. Livebirth after uterus transplantation from a deceased donor in a recipient with uterine infertility. The Lancet 2018;392:2697–704 and The Independent. Baby girl born after mother receives transplanted womb from dead donor in US first https://www.independent.co.uk/news/world/americas/womb-transplant-baby-born-us-cleveland-uterus-donor-dead-a8999121.html, 2019. (22 July 2019, date last accessed). 10.1016/S0140-6736(18)31766-5 30527853

[3] Health Research Authority NHS Uterine Transplantation in the Human Setting. https://www.hra.nhs.uk/planning-and-improving-research/application-summaries/research-summaries/uterine-transplantation-in-the-human-setting/, 2015. (25 January 2019, date last accessed).

[4] BBC. First UK womb transplant ‘by end of 2018’. https://www.bbc.co.uk/news/health-44360786, 2018. (25 January 2019, date last accessed).

[5] Johannesson L, Dahm-Kähler P, Eklind S, et al. The future of human uterus transplantation. Women's Health 2014;10:455–67. 10.2217/whe.14.22 25259905

[6] Hellström M, El-Akouri RR, Sihlbom C, et al. Towards the development of a bioengineered uterus: comparison of different protocols for rat uterus decellularization. Acta Biomater 2014;10:5034–42. 25169258 10.1016/j.actbio.2014.08.018

[7] Womb Transplant UK. Why the need for womb transplants? http://wombtransplantuk.org/about/why, 2019 (4 March 2019, date last accessed).

[8] Fageeh W, Raffa H, Jabbad H, et al. Transplantation of the human uterus. Int J Gynecol Obstet 2002;76:245–51. 10.1016/s0020-7292(01)00597-5 11880127

[9] Fornalik H, Fornalik N Uterus transplantation: robotic surgeon perspective. Fertil Steril 2018;109:365. 29246556 10.1016/j.fertnstert.2017.10.038

[10] Brännström M, Dahm-Kähler P, Kvarnström N Robotic-assisted surgery in live-donor uterus transplantation. Fertil Steril 2018;109:256–7. 29395094 10.1016/j.fertnstert.2017.12.007

[11] Kisu I, Kato Y, Matsubara K, et al. Emerging problems in uterus transplantation. BJOG 2018;125:1352–6 3. 29869370 10.1111/1471-0528.15230

[12] Lefkowitz A, Edwards M, Balayla J The Montreal criteria for the ethical feasibility of uterine transplantation. Transpl Int 2012;25:439–47. 22356169 10.1111/j.1432-2277.2012.01438.x

[13] Brännström M Current status and future direction of uterus transplantation. Curr Opin Organ Transplant 2018;23:592–7. 30138148 10.1097/MOT.0000000000000568

[14] Jones BP, Saso S, Yazbek J, et al. Uterine transplantation: past, present and future. BJOG 2016;123:1434–8. 26931179 10.1111/1471-0528.13963

[15] Russo NF The motherhood mandate. J Soc Issues 1976;3:143–53.

[16] Lotz M Commentary on Nicola Williams and Stephen Wilkinson: ‘Should uterus transplants be publicly funded?’. J Med Ethics 2016;42:570–1. 26960350 10.1136/medethics-2015-103230

[17] Petropanagos A Pronatalism, geneticism and ART. Int J Fem Approaches Bioethics 2016;10:119–47.

[18] Johannesson L, Järvholm S Uterus transplantation: current progress and future prospects. Int J Womens Health 2016;8:43–51. 26917976 10.2147/IJWH.S75635 PMC4751897

[19] Brännström M, Wranning CA, Altchek A Experimental uterus transplantation. Hum Reprod Update 2010;16:329–45. 19897849 10.1093/humupd/dmp049

[20] Kisu I, Banno K, Mihara M, et al. Current status of uterine transplantation in primates and issues for clinical application. Fertil Steril 2013;100:280–94. 23557761 10.1016/j.fertnstert.2013.03.004

[21] Milliez J Uterine transplantation FIGO committee for the ethical aspects of human reproduction and women’s health. Int J Gynecol Obstet 2009;106:270.

[22] Olausson M, Johannesson L, Brattgård D, et al. Ethics of uterus transplantation with live donors. Fertil Steril 2014;102:40–3. 24784936 10.1016/j.fertnstert.2014.03.048

[23] Shapiro ME, Ward FR Uterus transplantation: a step too far. AJOB 2018;18:36–7. 10.1080/15265161.2018.1478027 30040573

[24] Fageeh W, Raffa H, Jabbad H, et al. Case report – transplantation of the human uterus. Int J Gynecol Obstet 2002;76:245–51. 10.1016/s0020-7292(01)00597-5 11880127

[25] Chmel R, Novackova M, Janousek L, et al. Revaluation and lessons learned from the first nine cases of a Czech uterus transplantation trial: four deceased donor and five living donor uterus transplantations. Am J Transplant 2018;19:855–64. 30151893 10.1111/ajt.15096

[26] Brännström M, Johannesson L, Dahm-Kähler P, et al. The first clinical uterus transplantation trial: a six-month report. Fertil Steril 2014;101:1228–36. 24582522 10.1016/j.fertnstert.2014.02.024

[27] Testa G, Koon EC, Johannesson L, et al. Living donor uterus transplantation: a single center’s observations and lessons learned from early setbacks to technical success. Am J Transplant 2017;17:2901–10. 28432742 10.1111/ajt.14326

[28] Jones BP Personal communication. 2019.

[29] Catsanos R, Rogers W, Lotz M The ethics of uterus transplantation. Bioethics 2013;27:65–73. 21726265 10.1111/j.1467-8519.2011.01897.x

[30] Mertes H, Van Assche K UTx with deceased donors also places risks and burdens on third parties. AJOB 2018;18:22–4. 10.1080/15265161.2018.1478029 30040566

[31] Guntram L, Williams NJ Positioning uterus transplantation as a ‘more ethical’ alternative to surrogacy: exploring symmetries between uterus transplantation and surrogacy through analysis of a Swedish government white paper. Bioethics 2018;32:509–18. 30048000 10.1111/bioe.12469 PMC6221143

[32] Mumtaz Z, Levay A Ethics criteria for uterine transplants: relevance for low-income, pronatalistic societies? J Clinic Res Bioeth 2012;S1-004:1–5.

[33] Caplan AL Moving the womb. Hastings Cent Rep 2007;37:18–20. 17649898 10.1353/hcr.2007.0036

[34] Bruno B, Arora KS Uterus transplantation: the ethics of using deceased versus living donors. AJOB 2018;18:6–15. 10.1080/15265161.2018.1478018 PMC6296249 30040550

[35] Williams NJ Deceased donation in uterus transplantation trials: novelty, consent and surrogate decision making. AJOB 2018;18:18–20. 10.1080/15265161.2018.1478043 30040583

[36] Williams NJ Should deceased donation be morally preferred in uterine transplantation trials? Biothics 2016;30:415–24. 10.1111/bioe.12247 PMC4934926 26833553

[37] Wilkinson S, Williams NJ Should uterus transplants be publicly funded? J Med Ethics 2016;32:559–65. 10.1136/medethics-2015-102999 PMC5013100 26670671

[38] Api M, Boxa A, Ceyhan M Could the female-to-male transgender population be donor candidates for uterus transplantation? Turk J Obstet Gynecol 2017;14:233–7. 29379666 10.4274/tjod.55453 PMC5780567

[39] Allyse M, Hatem A, Christos C, et al. American Society for Reproductive Medicine position statement on uterus transplantation: a committee opinion. Fertil Steril 2018;110:605–10. 30196945 10.1016/j.fertnstert.2018.06.017

[40] National Health Service Commissioning Board and Clinical Commissioning Groups (Responsibilities and Standing Rules) (Amendment) Regulations 2017.

[41] Fertility Fairness. Freedom of information data. http://www.fertilityfairness.co.uk/wp-content/uploads/2018/10/England-FertilityFairness_FOI_2018.pdf, 2018 (12 February 2019, date last accessed).

[42] Note that these same arguments also apply to the provision of uterine transplant in non-publicly funded systems.

[43] Sandman L The importance of being pregnant: on the healthcare need for uterus transplantation. Bioethics 2018;32:519–26. 30318621 10.1111/bioe.12525

[44] Daar J, Klipstein S Refocusing the ethical choices in womb transplant, peer commentary reviewing John A. Robertson, Other women’s wombs: uterus transplant and gestational surrogacy. J L Biosci 2016;3:383–8. 10.1093/jlb/lsw011 PMC5033439 27774233

[45] Arora KS, Blake V Uterus transplantation: ethical and regulatory challenges. J Med Ethics 2014;40:396–400. 23760727 10.1136/medethics-2013-101400

[46] Balayla J Public funding of uterine transplantation. J Med Ethics 2016;42:568–9. 26858308 10.1136/medethics-2015-103232

[47] Robertson JA Is there a right to gestate? J L Biosci 2017;4:630–6. 10.1093/jlb/lsx010 PMC5965490 29868191

[48] Favre-Inhofer A, Rafii A, Carbonnel M, et al. Uterine transplantation: review in human research. J Gynecol Obstet Hum Reprod 2018;47:213–21. 29574054 10.1016/j.jogoh.2018.03.006

[49] Alghrani A Uterus transplantation in and beyond cisgender women: revisiting procreative liberty in light of emerging reproductive technologies. J L Biosci 2018;5:301–28. 10.1093/jlb/lsy012 PMC6121040 30191067

[50] Jones BP, Williams NJ, Saso S, et al. Uterine transplantation in transgender women. BJOG 2018;126:152–6. 30125449 10.1111/1471-0528.15438 PMC6492192

[51] Bayefsky MJ, Berkman BE The ethics of allocating uterine transplants. Camb Q Healthc Ethics 2016;25:350–65. 26864991 10.1017/S0963180115000687

[52] Human Fertilisation and Embryology Act 1990, s.13(5) 11651088

[53] Human Fertilisation and Embryology Authority Code of Practice (2018) 9th edn, para 8.14.

[54] Jackson E Conception and the irrelevance of the welfare principle. Mod L Rev 2002;65:176–203.

