# Corrigendum to: Ethical and policy issues raised by uterus transplants

**DOI:** 10.1093/bmb/ldaa020

**Published:** 2020-07-08

**Authors:** Laura O’Donovan, Nicola Jane Williams, Stephen Wilkinson

**Affiliations:** Department of Politics, Philosophy, & Religion, County South, Lancaster University, Lancaster, LA1 4YQ, United Kingdom; Department of Politics, Philosophy, & Religion, County South, Lancaster University, Lancaster, LA1 4YQ, United Kingdom; Department of Politics, Philosophy, & Religion, County South, Lancaster University, Lancaster, LA1 4YQ, United Kingdom

The first version of this article did not include its funding support from the Leverhulme Trust. This has now been corrected.

